# Oral and Subcutaneous Immunization with a Plant-Produced Mouse-Specific Zona Pellucida 3 Peptide Presented on Hepatitis B Core Antigen Virus-like Particles

**DOI:** 10.3390/vaccines11020462

**Published:** 2023-02-17

**Authors:** Khadijeh Ghasemian, Inge Broer, Jennifer Schön, Richard Killisch, Nadine Kolp, Armin Springer, Jana Huckauf

**Affiliations:** 1 Department of Agrobiotechnology and Risk Assessment for Bio and Gene Technology, Faculty of Agricultural and Environmental Sciences, University of Rostock, 18059 Rostock, Germany; 2 Department of Reproduction Biology, Leibniz Institute for Zoo and Wildlife Research (IZW), 10315 Berlin, Germany; 3 BIOSERV, Analytik und Medizinprodukte GmbH, 18059 Rostock, Germany; 4 Medical Biology and Electron Microscopy Center, Rostock University Medical Center, 18057 Rostock, Germany

**Keywords:** contraceptive vaccine, zona pellucida 3 peptide, chimeric virus-like particles, plant molecular farming, oral vaccine, *Nicotiana benthamiana*

## Abstract

A short mouse-specific peptide from zona pellucida 3 (mZP3, amino acids 328–342) has been shown to be associated with antibody-mediated contraception. In this study, we investigated the production of mZP3 in the plant, as an orally applicable host, and examined the immunogenicity of this small peptide in the BALB/c mouse model. The mZP3 peptide was inserted into the major immunodominant region of the hepatitis B core antigen and was produced in *Nicotiana benthamiana* plants via *Agrobacterium*-mediated transient expression. Soluble HBcAg-mZP3 accumulated at levels up to 2.63 mg/g leaf dry weight (LDW) containing ~172 µg/mg LDW mZP3 peptide. Sucrose gradient analysis and electron microscopy indicated the assembly of the HBcAg-mZP3 virus-like particles (VLPs) in the soluble protein fraction. Subcutaneously administered mZP3 peptide displayed on HBcAg VLPs was immunogenic in BALB/c mice at a relatively low dosage (5.5 µg mZP3 per dose) and led to the generation of mZP3-specific antibodies that bound to the native zona pellucida of wild mice. Oral delivery of dried leaves expressing HBcAg-mZP3 also elicited mZP3-specific serum IgG and mucosal IgA that cross-reacted with the zona pellucida of wild mice. According to these results, it is worthwhile to investigate the efficiency of plants producing HBcAg-mZP3 VLPs as immunogenic edible baits in reducing the fertility of wild mice through inducing antibodies that cross-react to the zona pellucida.

## 1. Introduction

Rodents cause economic, social, and environmental damage worldwide and can endanger the health of humans, companion animals, and livestock [1,2]. They can be one of the serious threats to food production in many parts of the world, such as Europe [3], Africa [4,5], Australia [6], and Asia [7,8]. It is necessary to manage the population of rodent pests with a species-specific and more humane approach to reduce their abundance below the damage threshold [9]. Fertility control has become an acceptable method of wildlife population management compared to conventional lethal methods such as culling, trapping, and poisoning that can threaten non-target species and the environment [10,11,12]. Immunocontraception, the prevention of conception through immunological methods, has been considered a potentially useful method, especially in multiparous species such as mice. Immunocontraception based on gamete-specific molecules involved in sperm–egg interactions is more suitable for achieving acceptable and efficient contraception [13,14,15]. The zona pellucida (ZP) is an extracellular glycoprotein matrix that surrounds growing mammalian oocytes and ovulated eggs and is an essential molecule in sperm–egg interaction and fertilization [13,16,17]. Immunization against mouse ZP proteins has been extensively investigated, and the potential of ZP as a target to limit mouse populations has been demonstrated [11,18,19].

Murine ZP is composed of three glycoproteins: ZP1, ZP2, and ZP3. Mouse ZP3, the primary sperm receptor and acrosome reaction inducer, plays a key role in fertilization and has been suggested as an immunocontraceptive antigen [20,21]. The essential role of the ZP3 glycoprotein in fertilization has been substantiated using ZP3-knockout female mice; ZP3-null female mice are completely infertile [22,23]. Recombinant murine ZP3 antigens have been shown to induce effective contraceptive responses in immunized mice [12,19,24]. Nevertheless, contraceptive vaccines should be effective, inexpensive, and readily applicable. An additional crucial requirement, especially for the field application of contraceptive vaccines, is the species-specificity of the vaccine, which cannot be addressed using protein-based antigens [13,17].

The development of peptide contraceptive antigens can improve the safety and specificity of vaccines [25], thereby allowing the oral application of vaccines for wildlife population control. Peptide-based contraceptive vaccines that target species-specific and functional regions of reproductive antigens can increase the target specificity and reduce the risk of side effects [26,27]. Amino acid residues on ZP3, which are poorly conserved between species, have been described to be involved in sperm binding, and antibodies against these amino acids interfere with sperm binding [28,29,30]. A mouse-specific ZP3 glycopeptide (mZP3), corresponding to amino acid residues 328–342 that contain epitopes (B- and T-cell epitopes) associated with antibody-mediated contraception, has been shown to induce contraceptive antibodies in different mouse strains, including wild mice [31,32,33]. Nevertheless, the immunogenicity of small peptide-based vaccines results in reduced efficacy [25,34]. To compensate for this, an immunogenic carrier molecule can be used to improve the immunogenicity of vaccines [35,36].

One method that has proven successful in carrying and delivering peptide epitopes is the use of virus-like particles (VLPs), which are non-infectious particles assembled from viral structural proteins [37]. In this study, the hepatitis B core antigen (HBcAg) was used to present the contraceptive mZP3 peptide. Several studies have described the strong immunogenicity of HBcAg VLPs [38,39,40]. HBcAg VLPs are promising carriers for enhancing vaccine immunogenicity [41]. The hepatitis B core (HBc) particles consist of 180 or 240 HBcAg monomers that form dimeric units. The dimers are able to self-assemble into two types of icosahedral particles, small or large, with T = 3 (90 dimers) or T = 4 (120 dimers) symmetry, respectively. The repetitive structure and the presence of immunogenic epitopes within HBcAg confer superior properties and make HBcAg a very strong immunogen [36,37,42]. Numerous foreign epitope sequences can be introduced into different regions of HBcAg without interfering with the VLP assembly. The major immunodominant region (MIR) or c/e1 loop of the core protein, located at the tip of spikes on the surface of assembled particles, is the most favorable site for epitope presentation [43,44]. Epitopes inserted at this site can be repetitively presented on the surface of particles; multimeric presentation of the displayed epitopes leads to a high level of immunogenicity [42,45]. Thus, the mZP3 peptide sequence was incorporated into the MIR to benefit from the immune-enhancing properties of the HBcAg VLPs.

Although the delivery of contraceptives by traditional methods (dart or manual injection) may be suitable for larger mammals, oral vaccine delivery is more promising for population management and control of small animals, such as mice, which are dispersed over a large area. Plants are considered to have the potential for the production and oral delivery of vaccines, which can be directly used as feed and eliminate the purification or processing of antigens [46,47]. Plant-made vaccines can be protected from degradation in the stomach through bioencapsulation in plant cells [48,49] and can induce systemic (IgG) and mucosal (IgA) immunity [50,51]. Plants offer advantages, such as scalability, safety, ease of storage, cost efficiency, and eukaryotic protein processing [52,53]. Moreover, since mZP3 is naturally glycosylated and glycosylation can affect the efficiency of recombinant ZP3-based antigens [24], plants could be a suitable system for the expression of glycosylated mZP3. *Agrobacterium*-mediated transient transformation of plants provides a method for rapid and large-scale production of recombinant proteins in plants [54]. *Nicotiana benthamiana* is one of the most efficient plant hosts and is well studied and commonly used to produce high levels of transiently expressed proteins [55,56]. Using the MagnICON transient expression system, high yields of recombinant proteins (up to 80% of the total soluble protein) have been achieved within a few days in *N. benthamiana* [57,58].

In the present study, we aimed to produce HBcAg particles displaying the contraceptive mZP3 peptide in *N. benthamiana* using a MagnICON-based transient expression system, as a fast system for proof of concept. Because immunization experiments in wild mice are problematic, the immunogenicity of plant-produced HBcAg-mZP3 was investigated in BALB/c mice to produce anti-mZP3 antibodies, which were used in immunohistochemistry on ovarian sections from wild mice to assay antibody–oocyte interaction. Plant-produced HBcAg-mZP3 was used in the parenteral and oral immunization of female mice to evaluate the systemic and mucosal immunogenicity of HBcAg-mZP3 in immunized mice.

## 2. Materials and Methods

### 2.1. Construction of HBcAg-mZP3 Expression Vector

The full-length monomeric HBcAg gene was kindly provided by Dr. Hadrien Peyret (John Innes Center, UK). BsaI restriction sites were introduced at the 5′ and 3′ ends through amplification of the HBcAg gene, and the PCR fragment was cloned into the pJET1.2 cloning vector (CloneJET PCR Cloning Kit; Thermo Fisher Scientific, Braunschweig, Germany). The insert sequence, mZP3 peptide CSNSSSSQFQIHGPR corresponding to amino acid residues 328–342, was flanked by flexible GGGGS linkers. It was synthesized with codon usage optimized for expression in *N. benthamiana* (Eurofins MWG Operon, Ebersberg, Germany). The sequence was inserted into the MIR of HBcAg using SalI and AvrII restriction sites, which are the unique restriction sites at the c/e1 loop region of the HBcAg for ease of cloning. The resulting construct was named HBcAg-mZP3. To target HBcAg-mZP3 to the secretory pathway for facilitated glycosylation [59,60], the coding sequence of HBcAg-mZP3 was cloned into the TMV-based expression vector pICH31120 of the MagnICON system by BsaI restriction/ligation [61]. The MagnICON expression vectors were kindly provided by Nomad Bioscience GmbH (Halle/Saale, Germany). The identity of HBcAg-mZP3 was confirmed by sequencing.

### 2.2. Expression of HBcAg-mZP3 in N. benthamiana Leaves

The recombinant expression vector pICH-HBcAg-mZP3 was introduced into *Agrobacterium tumefaciens* strain ICF320 by electroporation. The resulting transformed colonies (confirmed by colony PCR and restriction enzyme digestion) were propagated in Luria–Bertani (LB) medium supplemented with 50 μg/mL kanamycin and 50 μg/mL rifampicin and incubated overnight in a shaking incubator (220 rpm, at 28 °C). To prepare the main bacterial culture, two milliliters of the starter culture was diluted in 200 mL LB, containing the same antibiotics, and incubated overnight under shaking conditions at 28 °C. The *agrobacterium* cells were sedimented at 4560× *g* for 30 min, and the pellet was resuspended in infiltration buffer containing 10 mM 2-(N-morpholino) ethanesulfonic acid (MES), pH 5.8, 10 mM MgSO_4_, and 0.02% *v*/*v* Silwet Gold to a final OD_600_ of 0.15–0.2. The bacterial suspension was vacuum-agroinfiltrated into the leaves of 6–8 weeks old greenhouse-grown *N. benthamiana* plants as previously described [62].

### 2.3. Protein Extraction

Small-scale protein extraction was performed for protein expression and accumulation analysis. Infiltrated leaves were harvested on different days post-infiltration (dpi) and frozen at −80 °C. The frozen samples were lyophilized for 72 h using an Alpha 1–4 LD plus freeze-dryer (Martin Christ GmbH, Osterode, Germany) and then ground to fine powder. Protein extraction was performed using four protein extraction buffers to optimize the extraction of HBcAg-mZP3 VLPs: extraction buffer 1 (50 mM sodium phosphate, pH 7.4, 100 mM NaCl, 50 mM Tris-HCl, 10 mM ethylenediaminetetraacetic acid (EDTA)), extraction buffer 2 (50 mM Tris-HCl, pH 7.25, 150 mM NaCl, 2 mM EDTA, 0.1% *w*/*v* Triton X-100, 1 mM dithiothreitol (DTT)), extraction buffer 3, PBS buffer (100 mM NaCl, 10 mM KCl, 6.5 mM Na_2_HPO_4_, 2 mM KH_2_PO_4_, pH 7.2), and extraction buffer 4 (10 mM NaH_2_PO_4_-H2O, 300 mM NaCl, 25 mM Tris-HCl, pH 7.4) plus complete protease inhibitor cocktail (Roche Diagnostics GmbH, Mannheim, Germany). Dry leaf samples were homogenized in ice-cold extraction buffer using a Precellys 24 homogenizer (Bertin Instruments, France). The clarified extract was obtained by repeated centrifugation at 15,000× *g* for 15 min at 4 °C. The supernatant was recovered as the soluble (S) protein fraction. The insoluble protein (IS) fraction was extracted by boiling the pellet in 1X SDS sample buffer and centrifugation at 15,000× *g* for 15 min.

### 2.4. Purification of Virus-like Particles (VLPs)

For large-scale extraction, infiltrated leaves were harvested at 8 dpi, lyophilized, and pulverized as described above. Ground leaf material was homogenized in chilled extraction buffer 2 using a homogenizer (Polytron PT 2100, Kinematica AG, Switzerland) for 30 s at 30,000 rpm. Homogenized tissue was rotated at 4 °C for 30 min to increase solubility. The homogenate was centrifuged at 4600× *g* for 15 min, and the extract was clarified by repeated ultracentrifugation at 16,000× *g* for 30 min at 4 °C.

To purify the assembled particles, the clarified extract was subjected to sucrose gradient sedimentation as previously described [63]. Briefly, the plant extract was layered at the top of a 10–60% (*w*/*v*) sucrose density gradient (dissolved in 10 mM Tris-HCl pH 8.4, 120 mM NaCl) and centrifuged in a TH-641 swing-out rotor at 187,000× *g* for 2.5 h at 4 °C. The gradient was fractionated and analyzed by Western blotting. In the second approach, VLPs were purified using double-layered sucrose cushions as previously described [64]. Briefly, clarified protein extracts were loaded above 2 mL of 25% (*w*/*v*) and 0.3 mL of 70% (*w*/*v*) sucrose and centrifuged as above. After ultracentrifugation, eight fractions were recovered from the bottom of the tubes and assayed for HBcAg-mZP3 content and VLP assembly using Western blotting and electron microscopy.

### 2.5. SDS-PAGE and Western Blot Analysis

Samples containing HBcAg-mZP3 were subjected to 12% SDS-PAGE after denaturation at 95 °C for 7 min in loading buffer (10% glycerin, 150 mM Tris (pH 6.8), 3% SDS, 1% β-mercaptoethanol, and 2.5% bromophenol blue). The separated proteins were transferred to nitrocellulose membranes (GE Healthcare Europe GmbH, Solingen, Germany). Membranes were first blocked with 5% (*w*/*v*) non-fat milk powder in TBST (20 mM Tris, 150 mM NaCl, and 0.05% (*v*/*v*) Tween20, pH 7.6) for 2 h at 20–22 °C (room temperature (RT)). After three washes with TBST, the membranes were probed with rabbit anti-mZP3 primary antibody (Bioserv, Rostock, Germany) to detect the mZP3 component of the protein. Following another washing step, the membranes were further probed with horseradish peroxidase (HRP)-conjugated goat anti-rabbit secondary antibody (Dianova, Hamburg, Germany) diluted 1:2000 in blocking buffer for 1 h at RT. The signals were then developed using ECL chemiluminescence reagents and detected by exposing the membranes to Kodak Biomax light X-ray film (VWR; Darmstadt, Germany). Concanavalin A (Con A) lectin blot analysis was conducted as previously described [62], to visualize the putative glycosylation of plant-made mZP3.

### 2.6. ELISA

The expression pattern of HBcAg-mZP3 was examined by ELISA that detects the mZP3 peptide. Briefly, the plates were coated with plant protein extract diluted in carbonate buffer (pH 9.6) for 2 h at RT. The plates were washed thrice with PBS containing 0.05% Tween 20 (PBST) and blocked further with 1X RotiBlock blocking solution (Carl Roth GmbH + Co. KG, Karlsruhe, Germany) for 1 h at RT. A rabbit anti-mZP3 antibody (Bioserv) was used as the primary antibody and incubated for 2 h at RT. Following another washing step, the plates were treated with HRP-conjugated goat anti-rabbit secondary antibody (Dianova) diluted 1:2000 in PBS. The plates were developed with a tetramethylbenzidine (TMB) substrate, the reaction was stopped with 250 mM H_2_SO_4_, and the absorbance (OD) was measured at 450 nm. To eliminate non-specific OD values, the extract of leaves infiltrated with pICH31120 was used as a control. *N. benthamiana*-produced mZP3 with defined quantity [65] was used as a reference standard. The amount of HBcAg-mZP3 protein was estimated based on the ratio of its molecular weight to the mZP3.

### 2.7. Transmission Electron Microscopy (TEM)

The partially purified HBcAg-mZP3 VLPs were fixed in fixative buffer (1% paraformaldehyde, 2% glutaraldehyde in 0.1 M phosphate buffer, pH 7.3) and then subjected to negative staining with 2% uranyl acetate. The prepared samples were analyzed using a transmission electron microscope (EM 902A, Zeiss, Oberkochen, Germany) equipped with a tungsten cathode. Representative areas of the samples were analyzed at 80 kV accelerating voltage. Imaging was performed using a CCD camera (CCD-Sensor THX 7888A, 14 µm × 14 µm pixel size, 1024 × 1024 pixels per mm2) (Proscan, Scheuring, Germany). Image processing was performed using iTEM software (Olympus Soft Imaging Solutions GmbH, Münster, Germany).

### 2.8. Mouse Immunization and Specific Serum IgG and Fecal IgA Measurement

Animal experiments were conducted in accordance with German animal protection regulations, with the approval of Landesamt für Landwirtschaft, Lebensmittelsicherheit und Fischerei, Mecklenburg-Vorpommern, Germany (File No. 7221.3–1-073/17 and 7221.3–1-071/20–2).

Female BALB/c mice were used for the immunization studies. For systemic immunization, five mice were injected subcutaneously with partially purified plant-produced HBcAg-mZP3 containing 5.5 µg mZP3, and the control group received PBS. Mice were immunized thrice at three-week intervals. All doses contained 10% Polygen (MVP Lab) as an adjuvant. Blood samples were collected prior to the primary injection, three weeks after the first immunization, and two weeks after the second or third immunization to obtain serum samples. Sera were diluted to 1:10^5^ and subjected to ELISA.

To determine the mucosal immunogenicity of plant-produced HBcAg-mZP3, two groups of mice (*n* = 10) were administered 120 mg of lyophilized leaf material suspended in 1 mL PBS by gavage. The treatment group received leaves expressing HBcAg-mZP3 (containing approximately 190 µg mZP3), and the control group received pICH31120-infiltrated leaves. Serum was collected prior to primary oral immunization and 20 days after each immunization, diluted 1:100, and subjected to ELISA. Feces were collected before the first mucosal immunization and seven days after each immunization and stored at −80 °C until being assayed for antibodies secreted by the intestinal mucosa. Fecal pellets were suspended in five volumes (*w*/*v*) of PBS extraction buffer (500 μL buffer per 100 mg fecal material), vortexed, and left at RT for 1 h. The feces were homogenized using a Precellys 24 homogenizer (Bertin Instruments). The extract was clarified by repeated centrifugation at 32,000× g for 10 min at 4 °C. Fecal extract was collected and subjected to ELISA.

The anti-mZP3 IgG and IgA responses in serum and fecal samples were detected using ELISA. *N. benthamiana*-produced mZP3 was used to coat the plates. Plates were incubated with 1.5 µg mZP3 in carbonate/bicarbonate coating buffer (pH 9.6) for 2 h at RT and then blocked with 1% (*w*/*v*) bovine serum albumin (BSA) in PBS for 1 h at RT. Subsequently, the plates were treated with serum samples or fecal extracts diluted in PBS for 2 h at RT. To measure endpoint antibody titers, serially diluted samples were added to the wells. After washing, the plates were incubated with HRP-conjugated donkey anti-mouse IgG (Dianova) or IgA (SouthernBiotech, Birmingham, Alabama, USA) diluted 1:2000 in PBS for 1 h at RT. After color development in the presence of TMB, the absorbance was measured at 450 nm. In the subcutaneous IgG assay, ELISA data were normalized by subtracting the non-specific readings (cells coated with extract of pICH31120-infiltrated leaves).

### 2.9. Indirect Immunofluorescence

Mouse ovarian sections were obtained from BIOSERV Analytik (Rostock, Germany). The sections of a wild mouse were deparaffinized and subjected to antigen retrieval using sodium citrate buffer (10 mM sodium citrate, 0.05% Tween 20, pH 6.0). Sections were blocked with 10% goat serum blocking solution (Life Technologies, Frederick, Maryland, USA) at RT for 1 h, washed with PBS, and incubated in 1X mouse-on-mouse IgG blocking solution (Invitrogen, Thermo Fisher Scientific, Waltham, MA, USA) at RT for 1 h. Sections were incubated with a 1:20 dilution of serum samples or fecal extracts and kept at 4 °C overnight. The sections were washed and incubated with 10 µg/mL fluorescein isothiocyanate (FITC)-conjugated goat anti-mouse IgG (Invitrogen, Thermo Fisher Scientific) as the secondary antibody for 1.5 h at 37 °C. After washing, the slides were mounted with DABCO mounting medium (25 mg/mL DABCO, 90% glycerol, and 10% PBS, pH 8.5) and observed under a fluorescence microscope.

### 2.10. Statistical Analysis

The experimental data were analyzed using the IBM SPSS statistical software version 27. Means were compared using one-way analysis of variance (ANOVA). Duncan’s test was used as a post hoc test to measure specific differences between the means. Statistical significance was set at *p* < 0.05.

## 3. Results

### 3.1. Design and Cloning of the HBcAg-mZP3 Expression Vector

**Figure 1:**
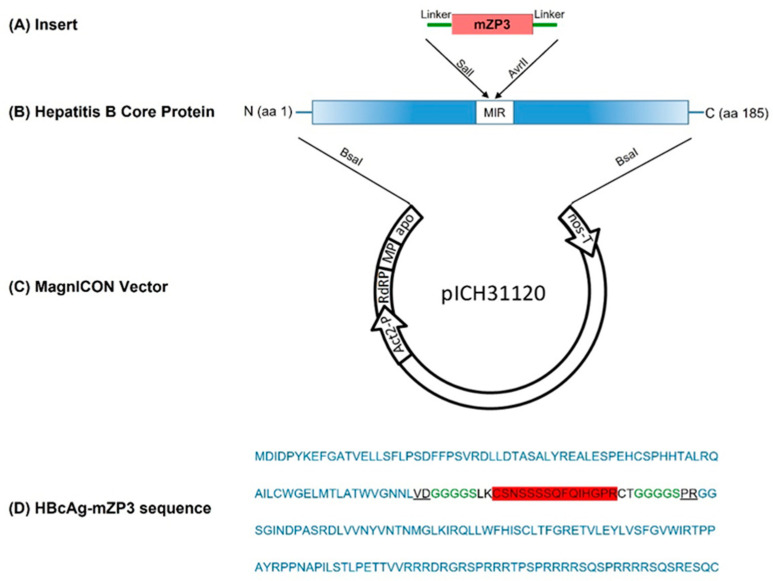
Schematic illustration of the recombinant vector, pICH-HBcAg-mZP3. Coding region for the mZP3-contraceptive epitope (**A**) inserted via flexible linkers (GGGGS) into the major immunodominant region (MIR) of full-length HBcAg (**B**). The MagnICON plant transient expression system was used in this study (**C**). Act2, Arabidopsis actin 2 promoter; RdRp, RNA-dependent RNA polymerase; MP, movement protein; apo: apple pectinase-apoplastidal targeting sequence; nos-T, nopaline synthase terminator. (**D**) Amino acid sequence of HBcAg-mZP3 construct. Amino acids of mZP3 peptide are shaded in red, flexible linkers are shown in green, and SalI and AvrII sites are underlined.

According to Peyret (2015), the coding sequence of the mZP3 contraceptive peptide was inserted into the c/e1 loop region of the HBcAg. The mZP3 antigenic peptide was linked to HBcAg via GGGGS flexible linkers on either side, predicted to not restrict particle assembly. The HBcAg-mZP3 construct was cloned into the MagnICON-based plant expression vector pICH31120 to target HBcAg-mZP3 to the secretory pathway (Figure 1). *Nicotiana benthamiana* plants were infiltrated with *Agrobacterium tumefaciens* containing HBcAg-mZP3.

### 3.2. Transient Expression of HBcAg-mZP3 in N. benthamiana Plants

**Figure 2:**
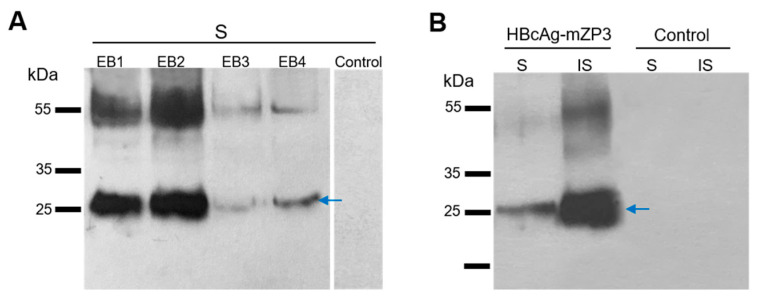
Western blot analysis of the HBcAg-mZP3 protein expressed in *N. benthamiana* leaves, identified in soluble (S) and insoluble (IS) fractions. (**A**) Effect of extraction buffer on soluble HBcAg-mZP3 extraction. Membrane probed with anti-mZP3 antibodies. (**B**) Detection and comparison of HBcAg-mZP3 protein in S and IS fractions extracted in EB2 condition. Extract from pICH31120-infiltrated leaves was used as control. Arrows indicate the monomeric form of HBcAg-mZP3. Blots cropped for clarity, for uncropped blots see Figure S2.

Following agroinfiltration, infiltrated leaves were harvested at 8 dpi, and HBcAg-mZP3 protein expression was investigated by Western blotting. Analysis of total soluble protein (TSP) extracted in four extraction buffers (EB) detected a protein band at the expected size of HBcAg-mZP3 using anti-mZP3 antibodies that specifically recognize mZP3. The results showed a monomeric form of the protein with a size of ~27 kDa and a higher protein band representing the dimer form of the protein. This protein was detected in leaves infiltrated with HBcAg-mZP3, whereas no protein band was found in extracts of leaves infiltrated with pICH31120, indicating the specificity of the HBcAg-mZP3 bands (Figure 2A). Analysis of equal amounts (30 µg) of TSP extracts showed that the highest amount of HBcAg-mZP3 was released into the TSP under EB2 conditions. However, a comparison between equal volumes of soluble and insoluble fractions from the EB2 condition showed that the majority of recombinant HBcAg-mZP3 remained insoluble (Figure 2B). More than 90% of the plant-produced HBcAg-mZP3 protein was estimated to be insoluble.

**Figure 3:**
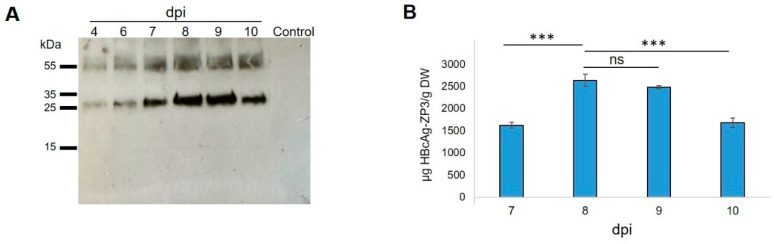
Western blot and ELISA analyses of HBcAg-mZP3 expression in agroinfiltrated *N. benthamiana* leaves from 4 dpi to 10 dpi. TSP was extracted from leaves on 4 dpi to 10 dpi. (**A**) Western blot analysis of protein extracts (20 µg TSP) probed with rabbit anti-mZP3 antibodies. Protein extracted from pICH31120-infiltrated leaves was used as control. dpi: days post infiltration. (**B**) Expression and accumulation of HBcAg-mZP3 were analyzed with ELISA of TSP extracted on 7–10 dpi. Error bars indicate standard deviation. *** indicates *p* values < 0.001. ns = non-significant difference (*p* < 0.5). See Figure S3 for uncropped blot. A Coomassie-stained gel of the leaf extract at 8 dpi is presented in Supplementary Material Figure S4.

The infiltrated leaves were evaluated by Western blotting for soluble HBcAg-mZP3 accumulation at different time points. Western blotting of TSP extracted from leaves on days 4–10 post infiltration was probed with an anti-mZP3 antibody. Although a positive signal was detected from 4 dpi, the highest accumulation of HBcAg-mZP3 was observed between 8 and 9 dpi (Figure 3A). At each time point, the leaf phenotype was also documented. The symptoms of leaf necrosis were visible from 6 dpi and gradually worsened, leading to severe wilting by 10 dpi (see Supplementary Figure S1 for phenotype of infiltrated leaf at 8 dpi). Furthermore, the expression and accumulation of recombinant HBcAg-mZP3 at different time points were quantified by ELISA using antibodies that recognized the mZP3 peptide. The expression of HBcAg-mZP3 reached the highest level at 8 dpi, with an average accumulation of 2633 µg/g leaf dry weight (LDW), corresponding to ~172 µg/g LDW mZP3 peptide, and then gradually decreased (Figure 3B). Based on the above observations, we set 8 dpi as the optimal harvest time for HBcAg-mZP3 expressed in *N. benthamiana*, using the MagnICON system.

### 3.3. Purification, Characterization, and Detection of Assembled HBcAg-mZP3 VLPs

**Figure 4:**
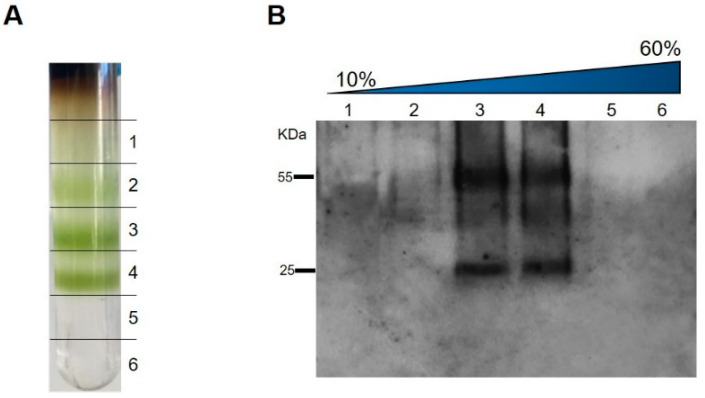
Gradient purification and detection of plant-produced HBcAg-mZP3. Total protein extract was subjected to a 10–60% sucrose gradient. (**A**) Photograph of an ultracentrifuge tube after ultracentrifugation. (**B**) Western blot analysis of sucrose gradient fractions. Sedimentation is left to right. An anti-mZP3 antibody was used to detect HBcAg-mZP3.

Leaves infiltrated with HBcAg-mZP3 were harvested at 8 dpi to purify the HBcAg-mZP3. Sucrose density gradients were applied to determine whether the expression of HBcAg-mZP3 in the plant led to the formation of VLPs. In the first purification method, extracts from HBcAg-mZP3-infiltrated leaves were sedimented on 10–60% sucrose gradients (Figure 4A), fractionated, and monitored by Western blotting. Analysis of the fractions from the sucrose gradient revealed a protein band corresponding to the size of HBcAg-mZP3. As shown in Figure 4B, the majority of HBcAg-mZP3 was distributed in 30–40% sucrose fractions, similar to what is known for assembled VLPs.

**Figure 5:**
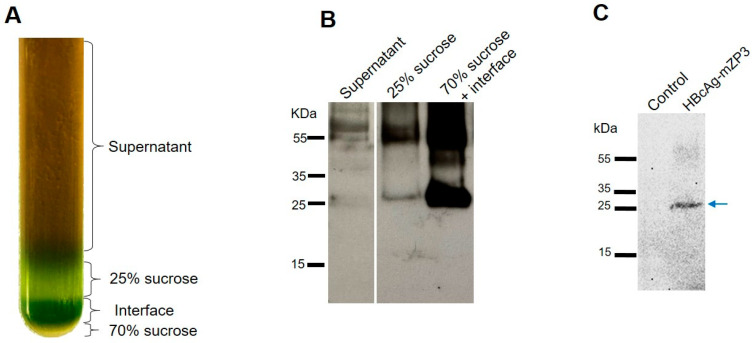
Partial purification of HBcAg-mZP3 VLPs using a double sucrose cushion. (**A**) Photograph of ultracentrifuge tube after ultracentrifugation. (**B**) HBcAg-mZP3 VLPs recovered from the interface and 70% fractions compared to other fractions. Western blots were probed with anti-mZP3 antibody to detect HBcAg-mZP3. (**C**) Lectin blot indicates binding of Con A with partially purified plant-produced HBcAg-mZP3. Lane 1, similarly prepared control sample (pICH31120-infiltrated leaves); lane 2, HBcAg-mZP3. Arrow indicates monomeric form of HBcAg-mZP3. Blots cropped for clarity, for uncropped blots see Figure S6.

In the second approach for sedimentation of HBcAg-mZP3, protein extracts containing HBcAg-mZP3 were loaded on a double sucrose gradient. After ultracentrifugation, a green band formed at the interface between the 25% and 70% sucrose layers (Figure 5A). Western blot analysis of the fractions showed that a major part of HBcAg-mZP3 sedimented towards the bottom of the tube (Supplementary Material Figure S5) and was mainly detected within the interface layer and the 70% sucrose layer below. The maximum HBcAg-mZP3 recovery was achieved when both the 70% fraction and the interface fraction were collected together (Figure 5B). A Coomassie-stained gel is presented in the Supplementary Material, Figure S4. These results strongly suggest that plant-produced HBcAg-mZP3 assembled into VLPs [64]. This method was used for preparing partially purified HBcAg-mZP3 for subsequent experiments.

Glycosylation of mZP3 was expected to be achieved by targeting HBcAg-mZP3 to the secretory pathway. Hence, a lectin blot assay was performed with peroxidase-conjugated concanavalin A (Con A), a carbohydrate-binding lectin, to investigate the possible glycosylation of plant-produced HBcAg-mZP3. The lectin blot of partially purified plant-produced HBcAg-mZP3 showed the reaction of HBcAg-mZP3 with Con A (Figure 5C), indicating the presence of sugar residues in the plant-produced HBcAg-mZP3, suggesting mZP3 glycosylation.

**Figure 6:**
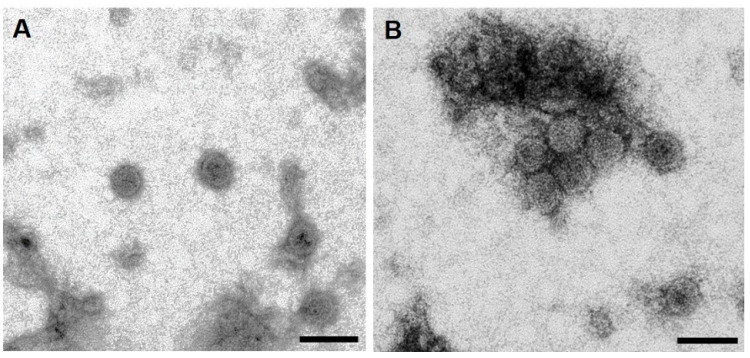
Electron microscopy of plant-produced HBcAg-mZP3 partially purified from leaf extract using sucrose gradients. TEM analysis of (**A**) HBcAg-mZP3 VLPs from 30–40% sucrose fractions, and (**B**) HBcAg-mZP3 VLPs from 70% sucrose fraction. Scale bars = 50 nm. A larger field image is presented in Supplementary Material Figure S7.

To confirm the formation of HBcAg-mZP3 VLPs, samples from the HBcAg-mZP3-rich fractions, 30–40% sucrose fractions collected from the sucrose density gradient and a 70% fraction from the double sucrose cushion, were subjected to negative staining and transmission electron microscopy (TEM). TEM observations confirmed that HBcAg-mZP3 produced in plants was successfully assembled into VLPs (Figure 6). The assembled HBcAg-mZP3 VLPs showed a typical shape and size similar to those documented in the literature for HBcAg VLPs.

### 3.4. Systemic Immunogenicity of Plant-Produced HBcAg-mZP3 in Mice

**Figure 7:**
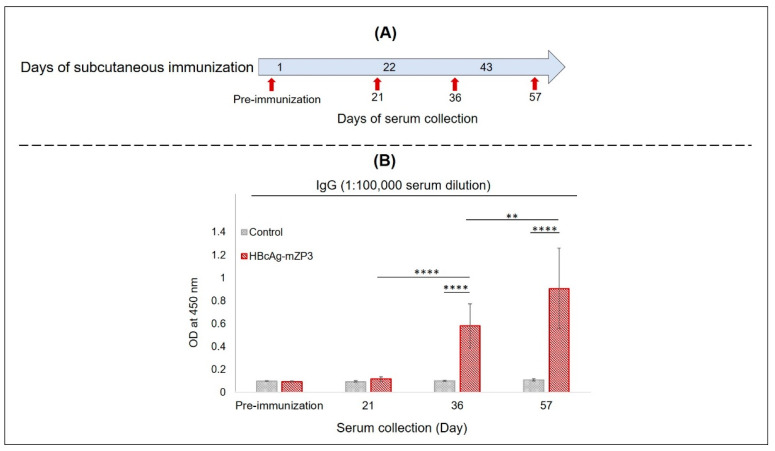
Evaluation of mean anti-mZP3 antibody responses in immunized BALB/c mice using ELISA. BALB/c mice subcutaneously immunized with plant-produced HBcAg-mZP3. (**A**)  Immunization and sampling schedule. (**B**) Anti-mZP3 IgG antibody levels measured in sera before immunization and after each immunization. Data presented as mean ± SD of absorbance values at 450 nm. ** *p* < 0.01 and **** *p* < 0.0001.

Because of the difficulties of maintaining wild-type mice in the laboratory, we examined the immunogenicity of mZP3 displayed on HBcAg VLPs in BALB/c mice. Subcutaneous immunization of mice was performed using partially purified plant-produced HBcAg-mZP3. The first group (*n* = 5) was immunized subcutaneously with three doses of HBcAg-mZP3 VLPs containing 5.5 μg mZP3 and 10% Polygen as an adjuvant, at three-week intervals. The control group was injected with saline buffer (PBS) with 10% Polygen. Serum samples were collected before immunization and on days 21, 36, and 57 (Figure 7A), and anti-mZP3 antibodies were measured in the sera from individual mice. In an indirect ELISA using a purified plant-produced mZP3 as the coating antigen, anti-mZP3 IgG antibodies were not detected in pre-immune serum or in sera after the prime dose (day 21). However, HBcAg-mZP3 elicited IgG antibody responses to mZP3 in immunized mice after the first booster dose, and these IgG antibody responses to mZP3 were significantly higher than those in the control group (*p* < 0.0001). A significant further boosting effect was observed after the last immunization dose, which caused significant (*p* < 0.01) elevations in the anti-mZP3 IgG responses, although there was variation between individual immunized mice (Figure 7B). While no anti-mZP3 response was observed throughout the immunization course in the control group, anti-mZP3 IgG was detectable in the sera of HBcAg-mZP3-immunized mice at serum dilutions over 1:10^5^ after booster doses. Pooled mice sera (*n* = 5) collected after the final subcutaneous immunization showed a mean IgG antibody titer of 1,000,000 (Supplementary Material, Figure S8A).

### 3.5. Mucosal Immunogenicity of Plant-Produced HBcAg-mZP3 in Mice

**Figure 8:**
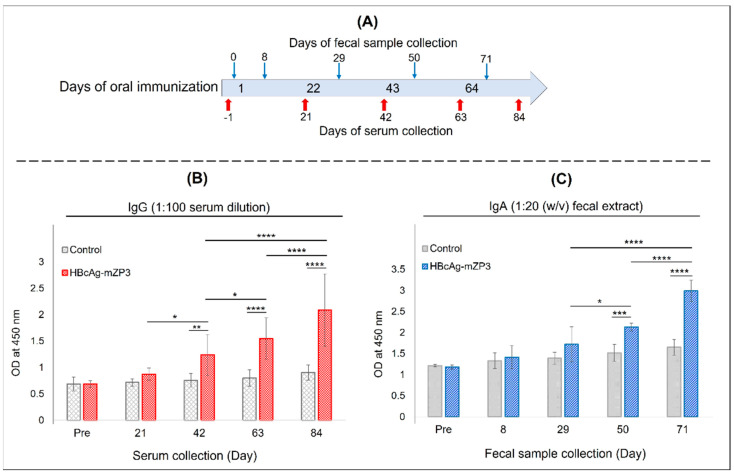
Evaluation of mean anti-mZP3 antibody responses in BALB/c mice orally immunized with plant leaves producing HBcAg-mZP3. (**A**)  Immunization and sampling schedule. (**B**) Anti-mZP3 IgG antibody levels measured in sera before immunization and after each immunization. (**C**) Anti-mZP3 IgA antibodies in feces extract measured in samples collected before and seven days after each oral immunization. Data presented as mean ± SD of absorbance values at 450 nm. * indicates *p* < 0.05; ** *p* < 0.01, *** *p* < 0.001, and **** *p* < 0.0001.

For the oral immunization of mice, 10 animals received a suspension of 120 mg lyophilized leaves expressing HBcAg-mZP3 (containing approximately 190 µg mZP3). Mice in the control group were administered similarly prepared control samples (pICH31120-infiltrated leaves). Sera and fecal samples were collected before immunization and after each immunization (Figure 8A) to measure the anti-mZP3 IgG and IgA antibodies. IgG anti-mZP3 antibodies were detected in sera after the second oral immunization, and the response increased significantly (*p* < 0.05) with the subsequent dose (Figure 8B). Furthermore, a higher boosting effect was observed after the last oral immunization, which significantly (*p* < 0.0001) increased specific anti-mZP3 IgG responses. The response detected in the control group was similar to that in the serum samples collected before the first immunization (Figure 8B). After the final oral immunization, the mean endpoint titers of anti-mZP3 IgG in the pooled sera (*n* = 10) reached the value of 10,000 (Supplementary Material, Figure S8B).

No significant IgA responses to mZP3 were detected in the fecal samples after the first two oral administrations of HBcAg-mZP3 compared to the pre-immunization and control groups (Figure 8C). However, the IgA response in fecal samples increased after the third oral dose of HBcAg-mZP3 and displayed a significant difference from that after the second dose (*p* < 0.05) and the control group (*p* < 0.001). As shown in Figure 8C, the fourth dose of the HBcAg-mZP3 oral vaccine markedly increased the mucosal IgA response in mice, which was significantly (*p* < 0.0001) higher than that of the third dose (8C). The responses detected in the control group may be due to the presence of antibodies against contaminating plant proteins [66]. The mean endpoint titer of anti-mZP3 IgA in pooled fecal extracts was ≥ 40 (*w*/*v*) and significant versus the control group (Supplementary Material, Figure S8C).

### 3.6. Binding of Antibodies to Native Zona Pellucida In Vitro

**Figure 9:**
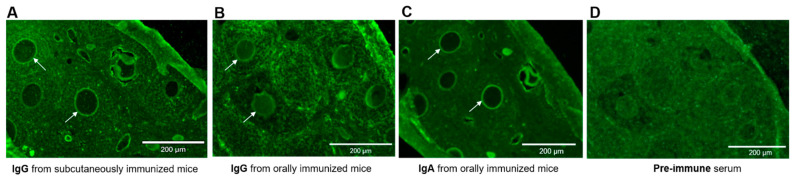
Indirect immunofluorescence microscopy to detect the binding of antibodies against HBcAg-mZP3 to ZP matrix. Sections from the same ovary were used. Wild mouse ovarian sections treated with (**A**) serum from subcutaneously immunized mice, (**B**) serum from orally immunized mice, and (**C**) fecal extracts from orally immunized mice. (**D**) Control experiments were performed with pre-immune serum. Arrows point to antibodies that reacted with the zona pellucida. Scale bars = 200 µm.

Serum IgG and mucosal IgA antibodies from HBcAg-mZP3-treated mice were used in an indirect immunofluorescence assay on wild mouse ovarian sections to evaluate the binding of antibodies to the ZP matrix. These observations showed that anti-mZP3 IgG antibodies generated in subcutaneously immunized female mice cross-reacted with the native ZP matrix in wild mouse ovaries (Figure 9A). Immune sera of orally immunized mice also reacted with the ZP of wild mice (Figure 9B). Furthermore, mucosal IgA antibodies from orally immunized mice specifically recognized and bound to the ZP of wild-type mice, and fluorescence was strongly observed around the oocytes (Figure 9C).

## 4. Discussion

The reduction of mouse fertility in the field requires an orally applicable, mouse-specific agent. The results described here provide a first step in this direction. They indicate that plants are a feasible platform for the production of sufficient quantities of putative mouse-specific mZP3 peptides. Plant-made mZP3 elicited ZP-specific antibody responses in immunized mice not only after subcutaneous administration, but also after oral administration. This study presented the oral immunogenicity of the mZP3 peptide for the first time. The induced antibodies cross-reacted with and bound to the wild mouse zona pellucida. Therefore, the efficacy of plant-produced mZP3 as an oral contraception vaccine to reduce the fertility of wild mice should be investigated.

Immunocontraception has been accepted as an alternative approach for managing overpopulated rodents and to address most concerns about the impact of rodenticides or other lethal methods of control on ecology and non-target species [3,15]. The mZP3 peptide produced in this study is not immunologically present in the ovaries of species such as guinea pigs, hamsters, cats, and dogs [33,67] and has been considered as a target for developing a mouse-specific contraceptive vaccine [26,28]. However, the species specificity of the vaccine to be administered orally should be verified. Ovarian damage or autoimmune oophoritis, which can be a concern of immunization with zona pellucida antigens [68], was not observed in this study in the presence of mZP3 peptide-induced autoantibodies.

The successful production of the small peptide mZP3 in the form of HBcAg-mZP3 in plants helps to address issues associated with the cost and scalability of vaccine production. We previously described the production of an immunogenic single mZP3 peptide in *N. benthamiana* plants [65]. As expected, the production of HBcAg-mZP3 protein resulted in a significant increase in mZP3 expression compared to the unconjugated mZP3 peptide. This is consistent with our earlier observations, which showed that mZP3 expression was significantly improved by fusion with a stable partner such as GFP [65]. HBcAg-mZP3 expression resulted in high production of the mZP3 peptide, similar to that produced by the polypeptide antigen mZP3–3, which contains three repeats of the mZP3 peptide [65]. The addition of DTT and Triton X-100 to the extraction buffer [63,69,70] increased the yield of soluble HBcAg-mZP3, and up to 2.63 mg/g dry weight HBcAg-mZP3 was detected. However, regardless of the buffer used, the HBcAg-mZP3 protein was mainly present in the insoluble fraction, which has also been reported for recombinant cytosolic HBcAg [71,72]. The formation of HBcAg-mZP3 virus-like particles was expected based on the distribution of HBcAg-mZP3 in particular sucrose gradient fractions [63,64,73] and was confirmed by electron microscopy. Despite the insertion of mZP3 into the MIR of HBcAg and targeting the protein to the secretory pathway, HBcAg-mZP3 assembled into VLPs similar to what has been observed for cytosolic wild-type HBcAg VLPs [63,73] and cytosolic [74] and ER-targeted [75,76] chimeric HBcAg VLPs. Hence, it can ensure the repetitive presentation of glycosylated mZP3 on the surface of assembled VLPs to elicit strong immune responses.

HBcAg was chosen to display mZP3 because several studies have reported the enhanced immunogenicity of small peptides conjugated to HBcAg VLPs [44,74]. Our results revealed that subcutaneous administration of plant-produced HBcAg-mZP3 elicited high humoral antibody responses against the mZP3 peptide compared to synthetic mZP3 [31] or *E. coli*-produced mZP3 peptides [26]. These results suggest that mZP3 can be readily displayed on the surface of particles and recognized by the immune system. Although the dose of mZP3 delivered in the form of HBcAg-mZP3 was approximately 10% of the HBcAg-free antigens used in our earlier study [65], the level of induced antibodies was higher than that of the single-peptide antigen, GFP-mZP3, and comparable to that of the polypeptide antigen, mZP3–3 (Supplementary Material, Figure S9). These results underscore the potential adjuvant effect of HBcAg VLP in enhancing the strong immune responses against mZP3. A reduction in the necessary dose to induce a strong immune response was expected because HBcAg VLPs have immune-enhancing properties and convey high immunogenicity to the heterologous epitope [41,44]; therefore, a lower dose of chimeric antigen (epitope) is usually sufficient [37,77]. Moreover, the high density of the mZP3 epitope repetitively displayed at the MIR, which is the most exposed and immunogenic region of particles, enhances immune responses against this heterologous epitope [39,44,78].

Transgenic plants can provide suitable formulations for the oral delivery of vaccines and reduce the costs of vaccine production significantly [51,52,79]. The results of this study indicate that repeated oral administration of plant cells containing HBcAg-mZP3 VLPs not only could evoke humoral immunity, but also was able to induce mucosal antibody responses against mZP3 in immunized mice. It has already been demonstrated that oral immunization of mice with a recombinant ZP3 protein could induce significant levels of IgG and IgA antibodies, leading to reduced fertility [80], and plant-produced HBcAg VLPs have also been shown to be capable of stimulating IgG and IgA responses in mucosal delivery [73,81]. This study is the first report of oral immunogenicity of the mZP3 peptide. Oral immunization with the small mZP3 in the form of HBcAg-mZP3 led to the induction of IgG and IgA antibodies that recognized the native ZP in wild mice. Hardy et al. [31] have shown that inducing antibodies cross-reacting with mZP3 resulted in reduced fertility in wild mice. Therefore, binding of induced antibodies to the native ZP of wild mice in vitro might reflect binding to the wild mouse ZP in vivo. Hence, further research is recommended to investigate the contraceptive efficacy of plants producing HBcAg-mZP3 VLPs as oral baits in wild mice.

Oral administration of the fourth dose of the plant tissue containing HBcAg-mZP3, without any additional adjuvants, induced a significant response to mZP3 compared with the third dose, which clearly indicated a boosting effect. It has been reported that oral contraceptive vaccines could be effective for a long time after multiple immunizations by inducing mucosal immunity in addition to systemic immunity [80,82]. Some reports have shown that the oral delivery of repeated or higher doses of antigens induces a significant and long-term immune response [83,84]. On the other hand, others supported less frequent and lower doses of oral antigens for inducing an active immune response [85,86] because higher doses or continuous oral administration of antigens can result in significantly stronger oral tolerance [87]. Therefore, the dose and frequency of HBcAg-mZP3 administration must be determined to establish an effective regime for inducing efficient immune responses.

In summary, we demonstrated the successful production of glycosylated mZP3 presented on HBcAg VLPs. We showed that subcutaneous and oral delivery of plant-produced HBcAg-mZP3 can stimulate anti-mZP3 antibodies in BALB/c mice. Both anti-mZP3 IgG and IgA antibodies showed reactivity and binding to the zona pellucida in the ovaries of wild mice. It is therefore likely that plant-produced mZP3 is capable of inducing zona pellucida-reactive antibodies, which may inhibit fertility in wild-type mice. Nevertheless, this has to be proven by the oral delivery of feed pellets containing plant-expressed HBcAg-mZP3 protein to wild mice. In addition, species specificity must be verified to ensure that the vaccine can be safely released into the environment without affecting non-target species.

## Data Availability

The datasets generated during this study are available from the corresponding author upon request.

## Supplementary Materials

The following supporting information can be downloaded at: https://www.mdpi.com/article/10.3390/vaccines11020462/s1, Figure S1: Phenotype of *N. benthamiana* leaves infiltrated with HBcAg-mZP3 at 8 dpi; Figure S2: Western blot analysis of the HBcAg-mZP3 protein expressed in *N. benthamiana* leaves, identified in soluble (S) and insoluble (IS) fractions. Figure S3. Western blot and ELISA analyses of HBcAg-mZP3 expression in agroinfiltrated *N. benthamiana* leaves from 4 dpi to 10 dpi. Figure S4: Coomassie-stained SDS-PAGE of extracts of *N. benthamiana* leaves. Figure S5: Partial purification of HBcAg-mZP3 VLPs using a double sucrose cushion. Figure S6: Partial purification of HBcAg-mZP3 VLPs using a double sucrose cushion and Lectin blot assay. Figure S7: Electron micrograph of HBcAg-mZP3 VLPs produced in *N. benthamiana*. Figure S8: Mouse mZP3-specific antibody titers. Figure S9: Comparison of serum antibody responses in mice immunized with mZP3 in the form of HBcAg-mZP3, GFP-mZP3–1, and mZP3–3 after the final boost injection.Figure S10: The endpoint titer ELISA data. The absorbance (OD) of each sample was read three times at 450 nm.
