# Supplementary material for: Oral and Subcutaneous Immunization with a Plant-Produced Mouse-Specific Zona Pellucida 3 Peptide Presented on Hepatitis B Core Antigen Virus-like Particles
Source: Vaccines (Basel). 2023 Feb 17;11(2):462. doi: 10.3390/vaccines11020462 (PMC9963689; doi:10.3390/vaccines11020462)
Supplement: Supplementary file 1 [file vaccines-11-00462-s001.zip › vaccines-2203354-supplementary.pdf]

Supplementary Materials

Oral and Subcutaneous Immunization with a Plant-Produced Mouse-Specific Zona Pellucida 3 Peptide Presented on Hepatitis B Core Antigen Virus-like Particles

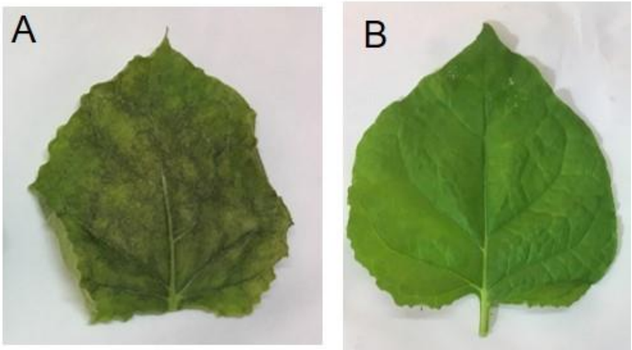

Figure S1. Phenotype of *N. benthamiana* leaves infiltrated with HBcAg-mZP3 at 8 dpi (**A**) compared to un-infiltrated leaf (**B**).

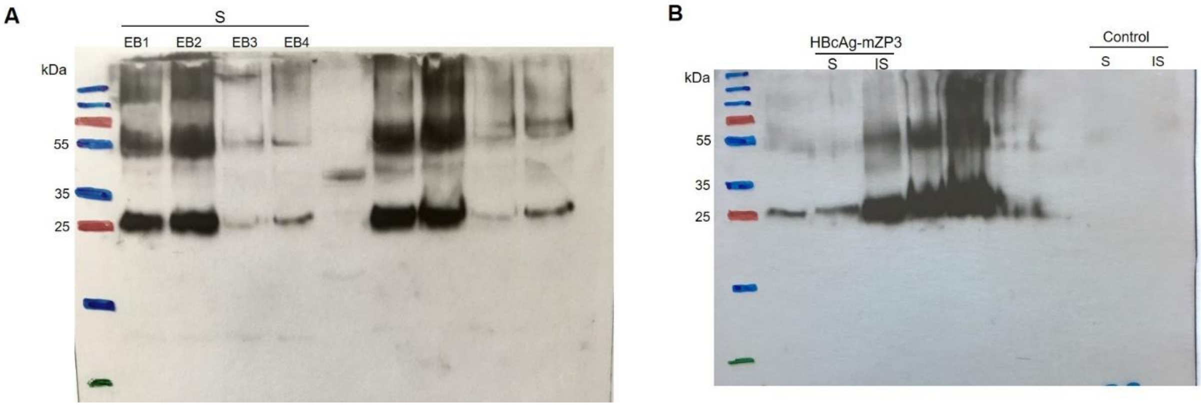

Figure S2. Western blot analysis of the HBcAg-mZP3 protein expressed in *N. benthamiana* leaves, identified in soluble (S) and insoluble (IS) fractions. Figure 2 was cropped from these western blot images.

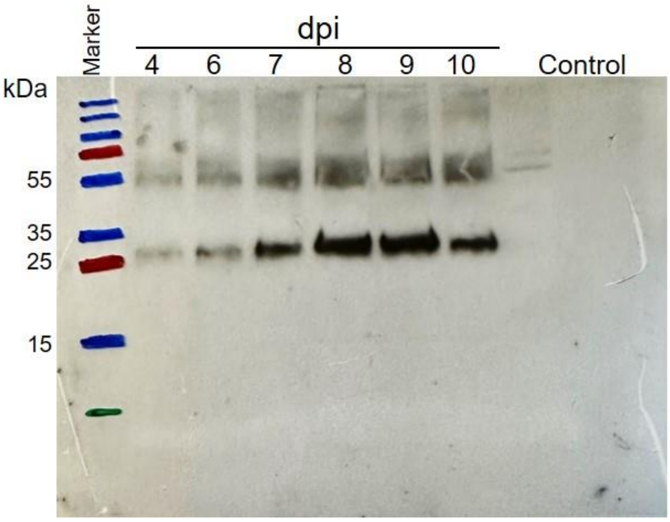

Figure S3. Western blot and ELISA analyses of HBcAg-mZP3 expression in agroinfiltrated *N. benthamiana* leaves from 4 dpi to 10 dpi. Figure 3 was cropped from this western blot image.

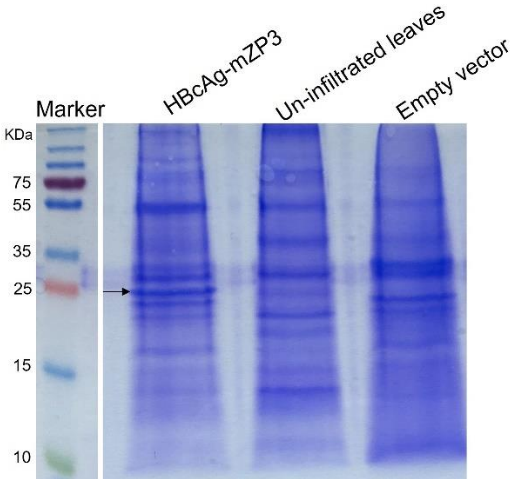

Figure S4. Coomassie stained SDS-PAGE of extracts of *N. benthamiana* leaves infiltrated with HBcAg-mZP3 (8 dpi), un-infiltrated leaves, and leaves infiltrated with pICH31120 empty vector. The protein band around 25 kDa could be HBcAg.

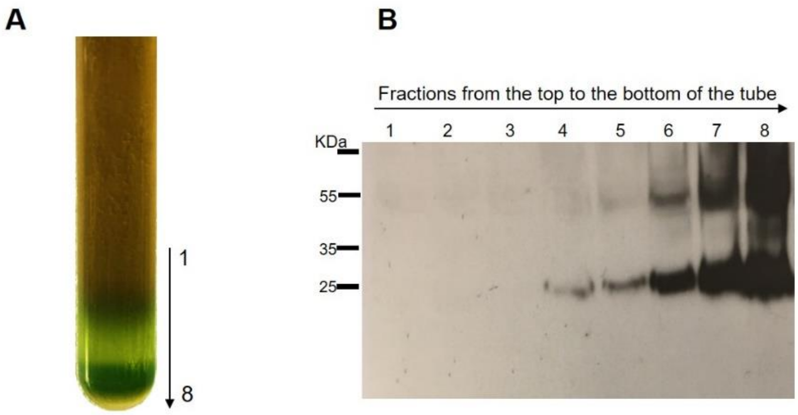

Figure S5. Partial purification of HBcAg-mZP3 VLPs using a double sucrose cushion. (A) Photograph of ultracentrifuge tube after ultracentrifugation. (B) Eight fractions (8 x 0.5 ml) were collected from the bottom of the tubes and analyzed by western blot.

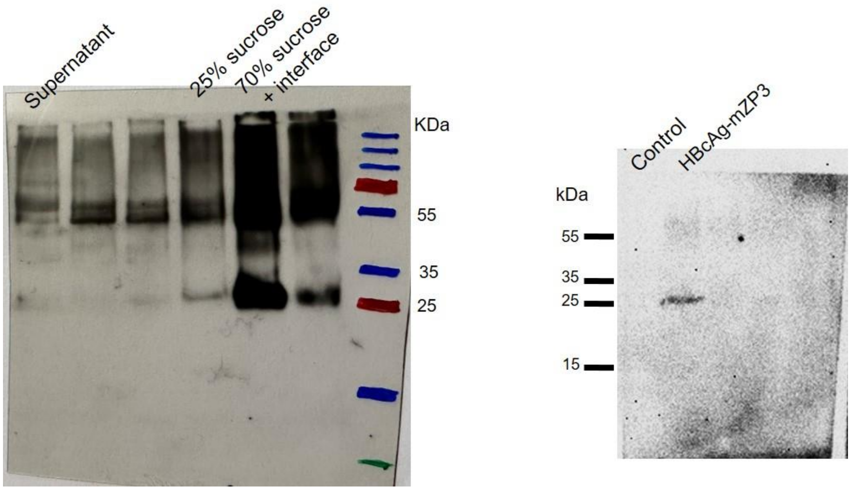

Figure S6. Partial purification of HBcAg-mZP3 VLPs using a double sucrose cushion and Lectin blot assay. Figure 5 was cropped from these western blot images.

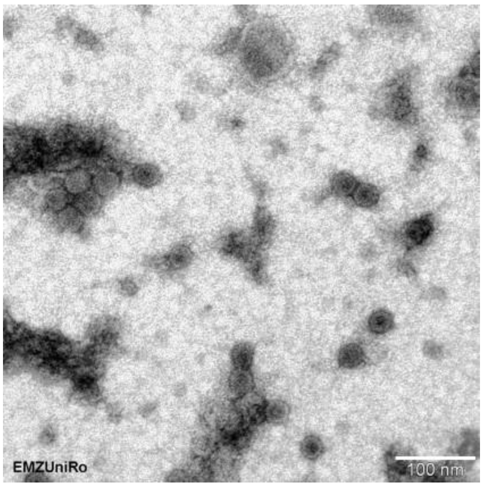

Figure S7. Electron micrograph of HBcAg-mZP3 VLPs produced in *N. benthamiana*. Bar = 100 nm.

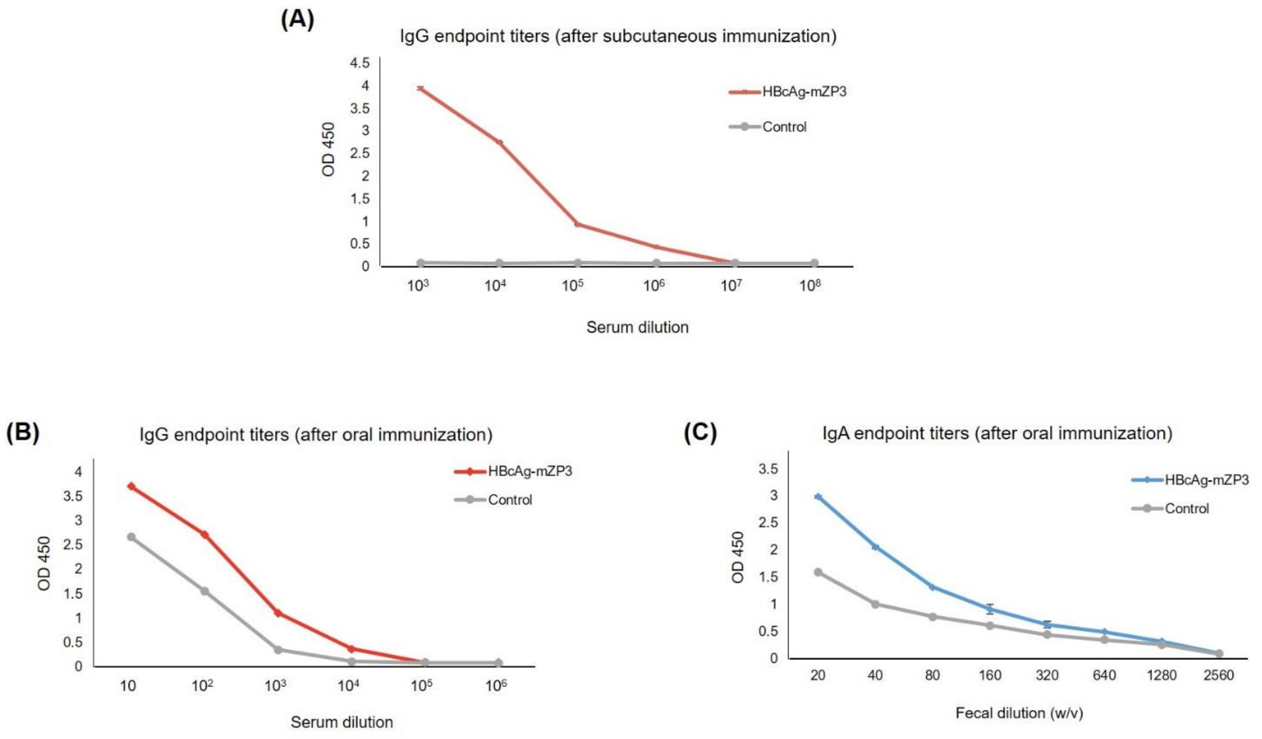

Figure S8. Mouse mZP3-specific antibody titers. (A) Pooled sera collected after the final subcutaneous immunization (n = 5), (B) pooled sera (n = 10), and (C) pooled fecal extracts (n = 10) collected after the final oral immunization, were analyzed for anti-mZP3 antibodies by endpoint titer ELISA. Error bars indicate the standard deviation of triplicate ELISA readings. The standard deviation of most data is small and invisible (See figure S10 for ELISA reading data).

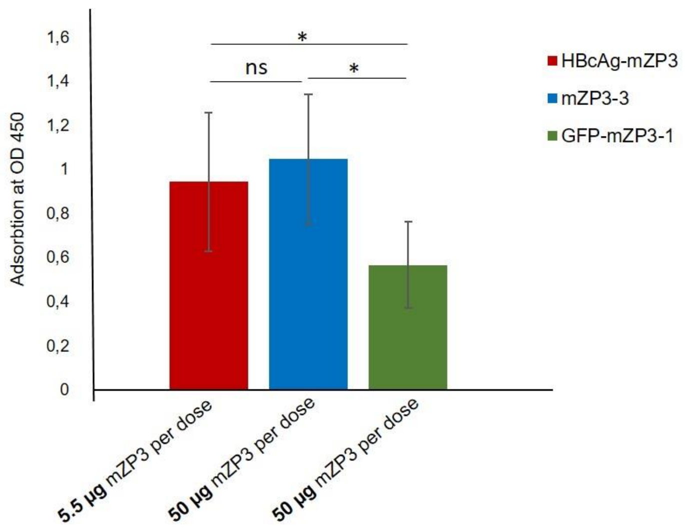

Figure S9. Comparison of serum antibody responses in mice immunized with mZP3 in the form of HBcAg-mZP3, GFP-mZP3-1 and mZP3-3 after the final boost injection. Absorbance values are shown for sera of each immunized group at a 1:100,000 dilution. Data are presented as the mean ± SD absorbance values at 450 nm. *$p < 0.05$; ns = non-significant difference ($p < 0.5$).

IgG, Subcutaneous immunization

		HBcAg-mZP3			Control		
		OD1	OD2	OD3	OD1	OD2	OD3
Serum dilution	10^3	3.914	3.948	3.977	0.086	0.084	0.084
	10^4	2.755	2.766	2.761	0.082	0.083	0.084
	10^5	0.924	0.940	0.932	0.085	0.087	0.083
	10^6	0.429	0.440	0.435	0.081	0.078	0.082
	10^7	0.084	0.082	0.083	0.079	0.078	0.080
	10^8	0.071	0.078	0.075	0.074	0.081	0.076

IgG, Oral immunization

		HBcAg-mZP3			Control		
		OD1	OD2	OD3	OD1	OD2	OD3
Serum dilution	10	3.709	3.698	3.704	2.702	2.620	2.667
	10^2	2.735	2.706	2.721	1.590	1.526	1.552
	10^3	1.123	1.090	1.107	0.338	0.373	0.356
	10^4	0.395	0.338	0.367	0.114	0.114	0.114
	10^5	0.096	0.094	0.095	0.096	0.087	0.092
	10^6	0.093	0.089	0.091	0.107	0.095	0.100

IgA, Oral immunization

		HBcAg-mZP3			Control		
		OD1	OD2	OD3	OD1	OD2	OD3
Fecal dilution	20	2.995	2.995	2.964	1.617	1.606	1.621
	40	2.040	2.090	2.065	1.005	1.022	1.011
	80	1.316	1.328		0.784	0.776	0.780
	160	0.829	1.004	0.917	0.625	0.606	0.616
	320	0.691	0.576	0.634	0.423	0.472	0.448
	640	0.499	0.501	0.500	0.347	0.363	0.355
	1280	0.325	0.327	0.321	0.268	0.262	0.265
	2560	0.108	0.115	0.112	0.088	0.107	0.098

Figure S10. The endpoint titer ELISA data. The absorbance (OD) of each sample was read three times at 450 nm.
